# Potential of PSMA-targeting radioligand therapy for malignant primary and secondary brain tumours using super-selective intra-arterial administration: a single centre, open label, non-randomised prospective imaging study

**DOI:** 10.1016/j.ebiom.2024.105068

**Published:** 2024-03-22

**Authors:** Ilanah J. Pruis, Pieter Jan van Doormaal, Rutger K. Balvers, Martin J. van den Bent, Anita A. Harteveld, Linda C. de Jong, Mark W. Konijnenberg, Marcel Segbers, Roelf Valkema, Frederik A. Verburg, Marion Smits, Sophie E.M. Veldhuijzen van Zanten

**Affiliations:** a Department of Radiology and Nuclear Medicine, Erasmus MC, Dr. Molewaterplein 40, 3015 GD, Rotterdam, the Netherlands; b Brain Tumour Centre, Erasmus MC Cancer Institute, Dr. Molewaterplein 40, 3015 GD, Rotterdam, the Netherlands; c Department of Neurosurgery, Erasmus MC, Dr. Molewaterplein 40, 3015 GD, Rotterdam, the Netherlands; d Department of Neurology, Erasmus MC Cancer Institute, Dr. Molewaterplein 40, 3015 GD, Rotterdam, the Netherlands; e Medical Delta, Delft, Huismansingel 4, 2629 JH, Delft, the Netherlands

**Keywords:** Malignant brain tumours, Prostate-specific membrane antigen, Theranostics, Radioligand therapy, Super-selective intra-arterial administration

## Abstract

**Background:**

The aim of this study was to provide quantitative evidence for the potential of PSMA-targeting radioligand therapy (RLT) as treatment approach for malignant brain tumours, and to explore whether tumour uptake could be enhanced by super-selective intra-arterial (ssIA)-administration.

**Methods:**

Ten patients (n = 5 high-grade glioma, n = 5 brain metastasis) received 1.5 MBq/kg [^68^Ga]Ga-PSMA-11 intravenously and, within 7 days, intra-arterially (i.e., selectively in tumour-feeding arteries), followed twice by PET-MRI at 90, 165 and 240 min post-injection. Patient safety was monitored for each procedure. Standardised uptake values (SUVs) were obtained for tumour, healthy-brain, salivary glands and liver. Tumour-to-salivary-gland (T/SG) and tumour-to-liver (T/L) uptake-ratios were calculated.

**Findings:**

No adverse events requiring study termination occurred. All patients showed uptake of [^68^Ga]Ga-PSMA-11 at the tumour site. Uptake was a median 15-fold higher following ssIA-administration (SUVmax median: 142.8, IQR: 102.8–245.9) compared to IV-administration (10.5, IQR:7.5–13.0). According to the bootstrap analysis, mean SUVmax after ssIA (168.8, 95% CI: 110.6–227.0) was well beyond the 95% confidence-interval of IV administration (10.5, 95% CI: 8.4–12.7). Uptake in healthy-brain was negligible, independent of administration route (SUVmean <0.1–0.1). Off-target uptake was comparable, resulting in more favourable T/SG- and T/L-ratios of 8.4 (IQR: 4.4–11.5) and 26.5 (IQR: 14.0–46.4) following ssIA, versus 0.5 (IQR: 0.4–0.7) and 1.8 (IQR: 1.0–2.7) for IV-administration.

**Interpretation:**

ssIA-administration is safe and leads to a median fifteen-fold higher radioligand uptake at the tumour site, therewith qualifying more patients for treatment and enhancing the potential of therapy. These results open new avenues for the development of effective RLT-based treatment strategies for patients with brain tumours.

**Funding:**

10.13039/100017296Semmy Foundation.

**Research in context**

**Evidence before this study**

PubMed was searched for peer-reviewed, original studies published in English using all relevant search terms, i.e., “radionuclide”, “tracer”, “radioligand”, “intra-arterial”, “IA”, “delivery”, “administration”, “theranostics”, “targeted radioligand therapy”, “brain”, “tumour”, “glioma”, “metastases” and “PET”. We found that there is a growing interest in the use of theranostics, meaning radioligands that can be used for molecular radioligand imaging and therapy (RLT), for brain tumour management. Several reviews have been published describing a wide range of potential molecular pathways and targets in brain tumours that may be useful for RLT. To date, a limited number of clinical studies have been performed, all applying conventional intravenous (IV) administration of the radioligand of interest. Unfortunately, results in terms of tumour uptake thus far temper the expectations with regard to efficacy. Intra-arterial (IA) administration in brain has been investigated extensively in the past for chemotherapeutics, but not for radioligands. Moreover, the benefit of IA administration was never before visualized nor quantified by imaging. Apart from one case report published by our own group, image-guided super-selective IA (ssIA) administration of radioligands in brain has never been described in literature.

**Added value of this study**

This is a prospective clinical imaging study assessing the feasibility, safety and benefit of ssIA (compared to IV) administration of a PSMA-targeting radioligand in patients with malignant brain tumours. In ten patients (n = 5 high-grade glioma, n = 5 brain metastasis) we showed by means of hybrid PET-MRI: 1) uptake of [^68^Ga]Ga-PSMA-11 in malignant brain tumours, which stably retained up to (at least) 240 min post-injection, while not engaging healthy brain, and 2) a median fifteen-fold higher uptake of [^68^Ga]Ga-PSMA-11 captured at the tumour site following ssIA administration, leading to standardised uptake values (SUVs) that well exceeded uptake in off-target dose-limiting organs such as the salivary glands, therewith qualifying more patients for treatment. Dosimetric modelling showed that, through the ssIA approach, multi-cycle RLT has the potential to deliver high cumulative radiation doses locally to brain tumours.

**Implications of all the available evidence**

This study provides quantified evidence and modelled predictions for the potential of PSMA-based RLT for treatment of malignant brain tumours. ssIA administration results in higher initial and remaining activity at the tumour site (proof of concept), therewith enhancing the efficiency in terms of absorbed radiation doses and consequently widening the therapeutic window for patient selection.

## Introduction

Malignant brain tumours are associated with high morbidity and mortality. Despite advances in local and systemic therapies, no curative treatment exists and patients’ prognosis remains poor.^1^ Novel approaches to improve diagnosis and therapy are therefore urgently required.

We introduce a combined approach including 1) the use of a theranostic radioligand, meaning a ligand designed to selectively target unique properties of cancer cells (or its microenvironment) that can be labelled with different radionuclides, either for diagnostic imaging or for treatment. And 2) intra-arterial administration of this compound, super-selectively (ssIA) into tumour feeding arteries. Our hypothesis is that the potential of theranostics for patients with brain tumours can be increased by local administration.

To date theranostic strategies are not yet widely applied for malignant brain tumours, but gain wide interest given the growing knowledge on molecular characteristics of brain tumours and rapid emergence of novel ligands. Radioligands for imaging (using e.g., fluor-18 or gallium-68) or therapy (using e.g., lutetium-177 or actinium-225) are increasingly being studied.^2^ Prostate specific membrane antigen (PSMA) is a ligand used for targeted radioligand therapy (RLT) in patients with prostate cancer. This therapy shows unprecedented success, with complete remissions, even in patients with widely disseminated disease who were formerly designated to palliative care.^3^ In recent studies, high PSMA expression was also found in other solid tumours including high-grade glioma (HGG) and brain metastasis (BM), particularly on endothelial cells of neovasculature.^4^ First PSMA-based PET studies in patients with brain tumours have shown generally comparable, but moderate uptake of various radioligands at the tumour site.^4^, ^5^, ^6^, ^7^ The first reports on PSMA-based RLT for patients with malignant brain tumours showed little side-effects, however, no improvement of survival has been documented yet; likely because of the observed limited tumour uptake following intravenous (IV) administration.^6^^,^^8^, ^9^, ^10^, ^11^, ^12^, ^13^, ^14^

IA administration, facilitating direct delivery of high, undiluted concentrations of pharmaceutical compounds, can be used to enhance local uptake at the tumour site. IA administration of chemotherapeutics in malignant brain tumours was first introduced in 1964 and has been studied extensively since. Recent Phase 1 and 2 clinical trials show therapeutic benefit, but also major toxicities.^15^^,^^16^ The use of radioligands here demonstrates an advantage in the ability to perform pre-therapeutic imaging, quantification and dosimetric modelling of target and off-target uptake. This allows selection of only those patients with a favourable balance between anticipated cytotoxic effects at the tumour site and systemic side-effects.

With the ever-advancing development of microcatheters, it is now possible to minimally-invasively reach even peripheral brain vasculature. This so-called endovascular approach is worldwide extensively performed for diagnostic angiographies and therapeutic interventions. Literature shows that morbidity is generally low with neurologic complications occurring in 2.6%, of which 0.1% were permanent.^17^ Apart from one case report,^18^ ssIA administration of a radioligand has never been performed in patients with malignant brain tumours.

In this prospective clinical (proof-of-concept) study we assess the feasibility, safety, and (quantified) benefit of ssIA compared to IV administration of a PSMA-based radioligand in patients with malignant brain tumours with the aim to improve the potential of future PSMA-based RLT strategies.

## Methods

### Ethics

Approval for this study was obtained from the Medical Ethics Committee at Erasmus MC (NCT05798273). Written informed consent was obtained from each patient in accordance with provisions of the declaration of Helsinki. Each study patient was allocated a unique research code to anonymise data. Source data were stored in a secured database. After inclusion was completed, the database was locked. Handling of data was in accordance to the Dutch General Data Protection Regulation.

### Patients

Patients were recruited during standard clinical visits to the department of neuro-oncology or neuro-surgery. Inclusion criteria were: radiologically-presumed and/or histologically-confirmed grade 2–4 glioma showing enhancement on post-contrast MRI, or brain metastasis/-es, planned for (re-)resection, age ≥18 years old, good clinical condition (Karnofsky performance status ≥70), and ability and willingness to provide written informed consent. The study protocol including a detailed description of the enrolment (in- and exclusion) criteria is published online (clinicaltrials.gov/study/NCT05798273). Full protocol and procedure details are available upon request.

### Procedures

[^68^Ga]Ga-PSMA-11 was prepared in-house using the commercially purchased precursor PSMA-11 (ABX, Radeberg, Germany). Patients received 1.5 MBq/kg [^68^Ga]Ga-PSMA-11 for IV and ssIA administration, in line with prostate cancer imaging guidelines.^19^ IV administration was performed following routine protocol. For ssIA administration, a sheath was placed in either of the groins to secure entrance to the arterial circulation, followed by a routine catheterisation procedure by a board-certified interventional neuroradiologist. Digital subtraction angiography (DSA) and 3D rotational CT was performed using Visipaque 270® (GE Healthcare, USA) as contrast agent, to determine the dominant tumour feeding arteries. After selective catheterisation of the main supplying artery/-ies [^68^Ga]Ga-PSMA-11 was administered by means of a pump, under supervision of a board-certified nuclear medicine physician and radiation protection officer. From the DSA images, data on the exact localization of the catheter(s), tumour enhancement, shunting and/or preferential flow were recorded for each patient.

### Adverse events

For safety reviews, a Safety Monitoring Charter was established and an independent Data Safety Monitor was appointed prior to study start (Supplementary Material). Adverse events were monitored during- and up to 24 h after all study procedures, and reported to the Medical Ethics Committee at Erasmus MC according to the protocol guidelines. No adverse events of special interest (AESI) were determined prior to study, since adverse effects from its use were not reported.

### Image acquisition

Scans were acquired on a 3.0T whole-body hybrid PET-MR system (Signa PET-MR, GE Healthcare, Chicago, Illinois, USA)) at 90 min (min; scan 1), 165 min (scan 2) and 240 min (scan 3) after IV and ssIA administration of [^68^Ga]Ga-PSMA-11 in order to assess tumour uptake kinetics and biodistribution over time. At all three time-points a single bed position (bp) PET scan of the brain was acquired for 15 min in “list mode” to enable dynamic (i.e., continuous) acquisition. Only the first scan was directly followed by a whole-body scan (from skull vertex to thighs) of 5 bp (each 3 min). Standard 4-tissue (air, lung, water, fat) MR DIXON based attenuation maps (MRAC) were acquired simultaneously; for the head this was combined with a standard MR ZTE scan that adds bone tissue to the MRAC. PET list-mode data were binned in one frame of respectively 30 min (scan 1, head and whole-body), 15 min (scan 2) and 20 min (scan 3) for reconstruction of static PET images. PET images were reconstructed using the in-house Q.Clear reconstruction algorithm, i.e., a block sequential regularised expectation maximisation algorithm using time-of-flight information, point-spread-functions and a beta value of 300. Corrections for attenuation, scatter and random coincidences were applied.

MRI sequences were acquired simultaneously at each time point according to the standard clinical protocol, including a T2-weighted (w) and T1w fast spoiled gradient-echo sequence of the head after a single bolus of contrast (0.1 mL/kg body weight; Gadobutrol, Gadovist 1.0 mmol/mL, Bayer AG, Berlin, Germany), reconstructed with a voxel size of 0.9 × 0.9 × 0.8 mm^3^.

### Image processing and analysis

#### Tumour uptake of [^68^Ga]Ga-PSMA-11

PET images were first visually inspected by a certified nuclear medicine physician (SEMVZ) and defined positive in case focal uptake of [^68^Ga]Ga-PSMA-11 at the tumour site exceeded local background (i.e., surrounding and contralateral healthy brain). To enable semi-quantitative analysis, standardised uptake values (SUVs) were calculated (Hermes Hybrid Viewer V4.0.0), according to standard clinical practice (SEMVZ, IJP). This corrects for differences in injected activities between ssIA versus IV administration, which resulted from a wider time window between production and injection, allowing for intra-patient comparisons. Following each procedure, the three sequential PET scans were semi-automatically co-registered with each other. Subsequently, a volume of interest (VOI) was manually drawn on PET around the tumour on scan one, and automatically copied to each of the other scans. From each scan, the highest measured uptake (i.e., SUVmax in Bq/mL) was retrieved to capture uptake in tumour, according to published recommendations for imaging and reporting of radioligand uptake on PET in glioma.^20^ Using SUVmax avoids partial volume effects by excluding non-tumour (i.e., background) uptake. Finally, the SUVmax of the three time-points were used to determine time activity curves (TACs) for both the IV and ssIA procedures, respectively.

PET images were also registered to simultaneously acquired T1w post-contrast and T2w MR images, in order to visually assess [^68^Ga]Ga-PSMA uptake patterns in relation to generally appreciated tumour characteristics, such as blood–brain barrier (BBB) disruption (T1 gadolinium enhancement) and non-enhancing infiltrating tumour/oedema (T2 hyperintensity).

#### Physiological uptake of [^68^Ga]Ga-PSMA-11

As a reference, mean uptake values (SUVmean) were obtained in contralateral healthy brain (i.e., background) by manually drawing a sphere with a similar size as the tumour area, such as done in comparable studies.^5^^,^^21^ In line with comparable imaging guidelines for glioma,^20^ tumour-to-background ratios were calculated by dividing SUVmax of tumour by SUVmean of background (TBRmax).

Physiological biodistribution of [^68^Ga]Ga-PSMA-11 was visually assessed from the PET scans. Subsequently, the SUVmean in the most relevant non-target organs was determined. The parotid glands are considered representative of all salivary glands,^22^ and designated as the dose-limiting organ for PSMA-targeting RLT.^23^ The liver is generally considered a suitable reference organ for intra-individual comparisons.^24^ In both parotid glands, spherical VOIs of 1 cm diameter were manually drawn on the PET followed by extraction to also cover the other time-points (similar as described for tumour), whereafter values were pooled. For the right liver lobe, a spherical VOI of 5 cm diameter was manually drawn on the whole-body PET, performed at the first time-point. Subsequently, tumour-to-salivary glands (T/SG) and tumour-to-liver (T/L) ratios were calculated by dividing the tumour SUVmax by the SUVmean of these organs.

#### Potential of PSMA-based RLT

Dosimetric modelling of the anticipated maximum absorbed radiation dose in Gray (Gy) per treatment cycle, with either lutetium-177 (using a model dose of 7400 MBq) or actinium-225 (using a model dose of 8 MBq), i.e., [^177^Lu]Lu-PSMA or [^225^Ac]Ac-PSMA-based RLT, was performed according to the EANM/MIRD guidelines for each patient and each administration route (MK, MSe, IJP).^23^ The contrast-enhancing area on MRI, commonly considered the most aggressive tumour area,^25^ was used to calculate the tumour volume.

The SUV in tumour at the three time points (90, 165 and 240 min p.i.) after both IV and ssIA administration of [^68^Ga]Ga-PSMA-11 were transformed into activity concentration uptake values. The uptake volume on PET, determined using a VOI using a SUV cut-off of 2.0 as based on the maximum uptake measured in background (i.e., healthy brain parenchyma), and body weight, were used to transform the SUV values into percentage of injected activity (%IA) per volume by: $\frac{\%IA\left(t\right)}{Volume}=\frac{100}{1000\times BW}\times SUV\left(t\right)$. The resulting TACs were fitted with exponential curves to enable estimation of the uptake kinetics past the last (240 min p.i.) time-point using Graphpad Prism (version 9.4.1, GraphPad Software, www.graphpad.com). Most of the TACs after IV administration showed a one-phase exponential association curve and most of the TACs after ssIA administration followed a single-exponential decay function.

The absorbed doses (D) achievable when using [^177^Lu]Lu-PSMA or [^225^Ac]Ac-PSMA were estimated from the TACs of [^68^Ga]Ga-PSMA-11 by following the MIRD-scheme as follows:^26^

(1) $$D\left(r_{t}\right)=\sum_{r_{s}}\tilde{A}\left(r_{s}\right)\times S\left(r_{t}\leftarrow r_{s}\right)$$

with *Ã* the time-integrated activity in source region *r*_*s*_ simulated for lutetium-177 or actinium-225 and *S(r*_*t*_*←r*_*s*_*)* the absorbed dose S-value in target region *r*_*t*_ per decay of lutetium-177 or actinium-225 in *r*_*s*_. Considering the short range of the beta- and alpha-particles from both isotopes only the self-dose S-values (*r*_*s*_ = *r*_*t*_) were considered. The time-integrated activity *Ã* is calculated by integration of the time–activity curves in tumour, multiplied by the lutetium-177 or actinium-225 decay function as follows:

(2) $$\tilde{A}\left(r_{s}\right)=\lim_{T\to \infty }\int_{0}^{T}A\left(0\right)e^{-\lambda_{biol}t}\times e^{-\lambda_{phys}t}dt=\lim_{T\to \infty }\frac{A\left(0\right)}{\lambda_{biol}+\lambda_{phys}}\left(1-e^{-\left(\lambda_{biol}+\lambda_{phys}\right)T}\right)$$

with *λ*_*biol*_ the biological clearance or uptake constant (equation (3)) and *λ*_*phys*_ the physical decay constant of lutetium-177/actinium-225. *A(0)* indicates the activity immediately after administration (*t = 0*), both *A(0)* and *λ*_*biol*_ are obtained by the curve fitting on the PET data. The advantage of IA administration can be estimated by integration over the period *T*_*eq*_ where the tumour uptake after IA administration exceeds the uptake after IV administration. From *T*_*eq*_ onwards assume only physical decay needs to be considered, equation (2) then becomes:

(3) $$\tilde{A}\left(r_{s}\right)=\frac{A\left(0\right)}{\lambda_{biol}+\lambda_{phys}}\left(1-e^{-\left(\lambda_{biol}+\lambda_{phys}\right)T_{eq}}\right)+\frac{A\left(0\right)}{\lambda_{phys}}e^{-\left(\lambda_{biol}+\lambda_{phys}\right)T_{eq}}$$

The S-values for the tumour volumes were determined with IDAC-dose 2.1,^27^ using the volumes determined using the post-contrast T1-weighted (T1w) image as reference (see below). Water-filled spheres were used as reference for the tumour S-values for lutetium-177 and for actinium-225 and its progeny. The relative biological effect (RBE) for the alpha-particles in the decay of actinium-225 and its progeny were assumed to be 5.

For tumour segmentation and semiautomatic calculation of the contrast-enhancing tumour volumes from post-contrast T1w images an in-house segmentation pipeline was used. To do so, pre-, and post-contrast T1w, T2w and fluid-attenuated inversion recovery scans were rigidly groupwise registered and subsequently registered using rigid registration followed by an affine registration to the ICBM 152 2009a nonlinear symmetric atlas, using Elastix (version 5.0.1).^28^^,^^29^ Automatic segmentation was performed using HD-GLIO, nnUNet task 1 and 82, and an extended version of nnUNet.^30^^,^^31^ Segmentation predictions were combined using the multi-label STAPLE algorithm.^32^ Resulting tumour segmentations were visually inspected and manually corrected by an independent researcher (IP, MSm) if needed, following which tumour volumes of the contrast-enhancing area were extracted by loading the images into ITK-SNAP version 3.6.0 (University of Pennsylvania and Utah, USA).^33^

### PSMA expression in tumour biopsies

Following the intra-arterial procedure, all patients underwent resection surgery the next day. Directly prior to resection, tumour biopsy samples were collected using per-operative neuronavigation. Tissue samples were formalin-fixed and paraffin-embedded. Tissue sections were immunostained with mouse anti-PSMA (clone: EP192, supplier: Cell Marque, catalogue number: 327R-18) and visually evaluated for PSMA-expression by experienced neuropathologists.

### Statistics

Statistical analysis was performed with Statistical Package for the Social Sciences (SPSS) version 24.0.0.1. (IBM Corporation, New York, USA). Baseline demographic and clinical data were obtained from medical records. Because of the relatively small sample size, median and interquartile ranges (IQR) were reported and paired sample t-tests with bootstrap 95% confidence intervals (CI) were performed to assess differences between IV and ssIA administration for: SUVmax of tumour, TBRmax, salivary glands (represented by parotid gland) and liver; T/SG and T/L ratios, and estimated maximum potential absorbed radiation doses (per tumour/per administration route). There is no allowance for multiplicity.

### Sample-size estimation

Sample size calculations were not performed, as baseline information regarding tumour uptake of [^68^Ga]Ga-PSMA-11 in this patient population was not known. Prior to study it was determined that inclusion would stop after n = 10 patients with a positive scan. The non-randomised set-up and sample size were justified based on feasibility, costs and patient burden.

### Role of funders

This work was supported by the Semmy Foundation (Stichting Semmy). The funder had no role in the study design, data collection, data analysis, interpretation or writing of the manuscript.

## Results

### Patients and data acquisition

**Fig. 1:**
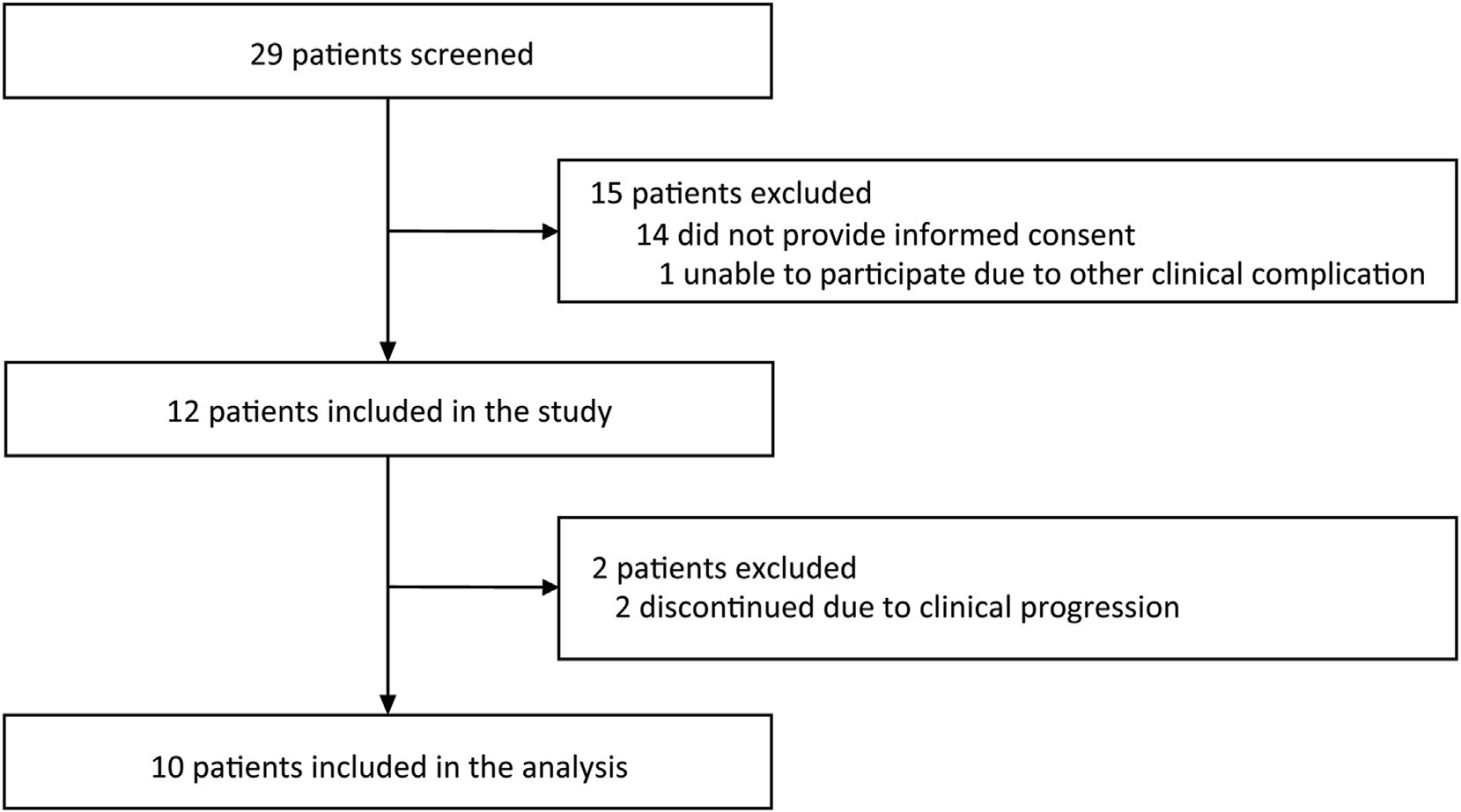
Flow chart of the study.

Ten patients with malignant primary or secondary brain tumours, including glioblastoma (n = 4), oligodendroglioma grade 3 (n = 1) and BM from lung- (n = 4) and breast carcinoma (n = 1) were included (Fig. 1, Table 1). The histological diagnosis was confirmed by post-operative tissue analysis in all patients. Patients received no concomitant brain tumour treatment during the inclusion period of the study. There were no missing data, apart from scan 1 of patient no. 3, due to the occurrence of a technical acquisition error. This did not influence the study analysis.

**Table 1:** Demographic data and tumour characteristics.

Patient no.	Sex	Age (years)	KPS	Diagnosis (grade, molecular subtype)	Primary or progression	Tumour location (lobe)	Previous treatment	TI to inclusion (weeks)
1	Male	73	80	Glioblastoma (grade 4, IDHwt)	Progression	Right parietal	S, RTx, TMZ	1
2	Male	61	90–100	Glioblastoma (grade 4, IDHwt)	Primary	Right cerebellar	None	1
3	Male	69	90	Glioblastoma (grade 4, IDHwt)	Progression	Right parieto-occipital	S, RTx, TMZ	2
4	Female	62	100	Glioblastoma (grade 4, IDHwt)	Progression	Right parietal	S, RTx, TMZ	4
5	Male	72	90	Oligodendroglioma (grade 3, IDHmt, 1p/19q codeletion)	Progression	Right frontal	S, RTx, PCV	5
6	Male	70	70–80	Brain metastasis from NSCLC	Progression	Right cerebellar	S, RTx	5
7	Male	57	80	Brain metastasis from NSCLC	Progression	Right frontal and temporal^a^	S, lorlatinib, RTx,	1
8	Female	63	80	Brain metastasis from breast cancer	Primary	Right frontal	None	3
9	Male	63	80–90	Brain metastasis from NSCLC	Progression	Left frontal and cerebellar^a^^,^^b^	RTx	2
10	Male	59	100	Brain metastasis from NSCLC	Primary	Right frontal and metastases^a^^,^^c^	RTx, C–P–P, sotorasib	19^d^

Abbreviations: C–P–P: carboplatin-pemetrexed-pembrolizumab; F: female; IDH: isocitrate dehydrogenase; KPS: Karnofsky Performance Scale; M: male; mt: mutant; NSCLC: non-small cell lung cancer, PCV: procarbazine, lomustine and vincristine; RTx: radiotherapy; S: surgery; TI: time interval in weeks between date of primary diagnosis or progressive disease and first study visit; TMZ: temozolomide; wt: wildtype.

a The frontal lesion was selectively approached during ssIA administration.

b Next to the frontal lesion, this patients had a small cerebellar lesion (<7 mm) on MRI suspect for metastasis.

c Next to the frontal lesion this patient had more than n = 20 infra- and supratentorial small lesions on MRI suspect for metastases.

d The relatively long time interval is explained by the fact this patient first received sotorasib treatment before tumour re-resection. Our study was introduced to the patient just prior to re-resection.

### Procedures

**Fig. 2:**
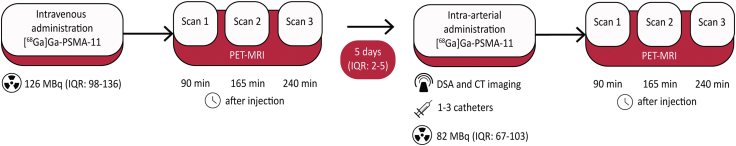
Schematic timeline of injection times and PET-MRI imaging protocol. Abbreviations: CT: computed tomography; DSA: digital subtraction angiography; IQR: interquartile range; MBq: megabecquerel; PSMA: prostate-specific membrane antigen.

Patient received a median of 126 (IQR: 98–136) MBq [^68^Ga]Ga-PSMA-11 after IV administration, and 82 MBq (IQR: 67–103) after ssIA administration. The median interval time between the IV and ssIA procedure was 5 days (IQR: 2–5). For ssIA administration a maximum of three catheter positions were used to locally administer the tracer (Table 2 and Fig. 2).

**Table 2:** Characteristics of ssIA administration procedure.

No.	Number of cath.	Localisation of catheter(s)	DSA enh.	DSA tumour shunting	DSA pref. flow
1	1	Right posterior parietal artery	No	No	No
2	3	Right superior cerebellar artery, left posterior inferior cerebellar artery and left anterior inferior cerebellar artery	Yes	Yes	No
3	1	Right P1-segment of posterior cerebral artery^d^	No	No	No
4	3	Right medial meningeal, right anterior and right medial cerebral artery	Yes	Yes	No
5	3	Right medial meningeal artery, right frontopolar artery and right superior division of medial cerebral artery	Yes	No	No
6	2	Right superior cerebellar artery and right posterior inferior cerebellar artery	No	No	No
7^a^	1	Right callosomarginal artery	Yes	No	No
8	2	Right prefrontal artery and right anterior frontopolar artery	Yes	No	No
9	2	Right pericallosal artery and right inferior division of medial cerebral artery	No	No	Yes
10	1	Right superior division of medial meningeal artery	No	No	No

Abbreviations: cath.: catheter; DSA: digital subtraction angiography; enh.: enhancement; pref.: preferential.

a Administration was performed in the P1 segment nearest to the dominant perforating arteries of mesencephalon.

### Adverse events

[^68^Ga]Ga-PSMA-11 was well tolerated by all patients following both IV and ssIA administration. One patient (no.1) experienced a serious adverse event (SAE) one day after ssIA administration, which consisted of partial weakness in the left arm and leg. A full stroke work-up was performed, including computed tomography (CT), CT angiography and CT perfusion of the brain, which showed no signs of ischaemia, haemorrhage, thrombus or perfusion deficits. The patient fully recovered without any intervention within a few days. A SAE was filed, including hypothesized possible causes for the partial weakness such as a late-induced vascular spasm, either resulting from ethanol present in the [^68^Ga]Ga-PSMA-11 solution (0.15 mL; 2 mL/8 mL with 50/50% (v/v) ethanol water), or from extravasation (i.e., leakage) of [^68^Ga]Ga-PSMA-11, and/or leakage of iodine/gadolinium-based contrast agents into the interstitial space. The SAE was reviewed by the ethical committee and deemed unrelated to study participation and consequently no adjustments were made to the study protocol. In this particular patient, as well as in two other patients (no. 3 and 4), the post-procedural scans showed [^68^Ga]Ga-PSMA-11 in brain/-vasculature at the location of the catheter tip (Supplementary Figure S1). The latter two patients did not experience any side-effects. No other SAEs were recorded between, during or after any of the study procedures in any of the patients.

### Tumour uptake of [^68^Ga]Ga-PSMA-11

**Fig. 3:**
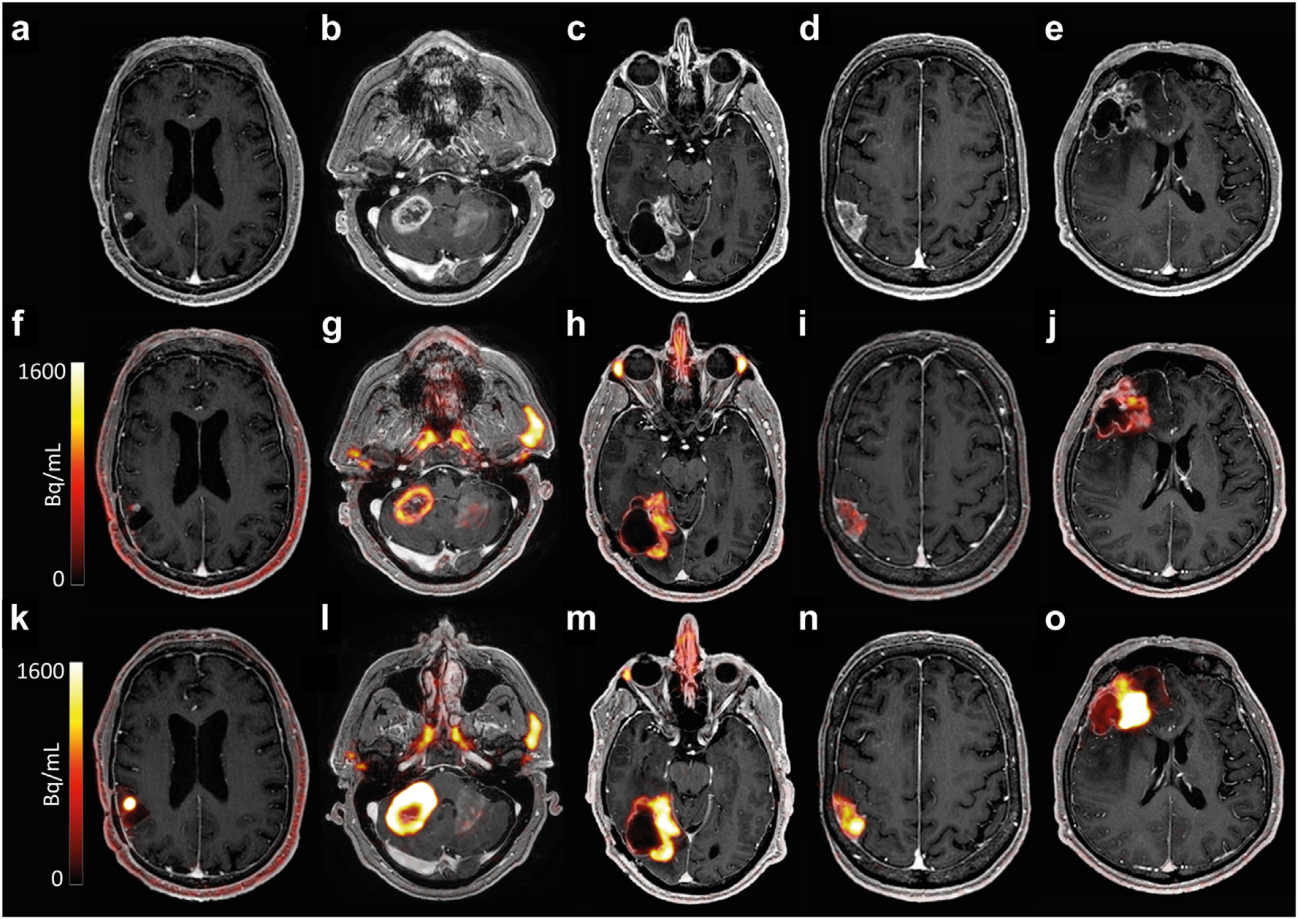
Uniformly scaled representative images of each patient with HGG (from left to right, no. 1–5) including a post-contrast T1w MR image (a–e), and fused [^68^Ga]Ga-PSMA-11 PET with post-contrast T1w MR images after IV (f–j) and ssIA administration (k–o). Abbreviations: HGG: high-grade glioma; IV: intravenous; PSMA: prostate-specific membrane antigen; ssIA: super-selective intra-arterial.

**Fig. 4:**
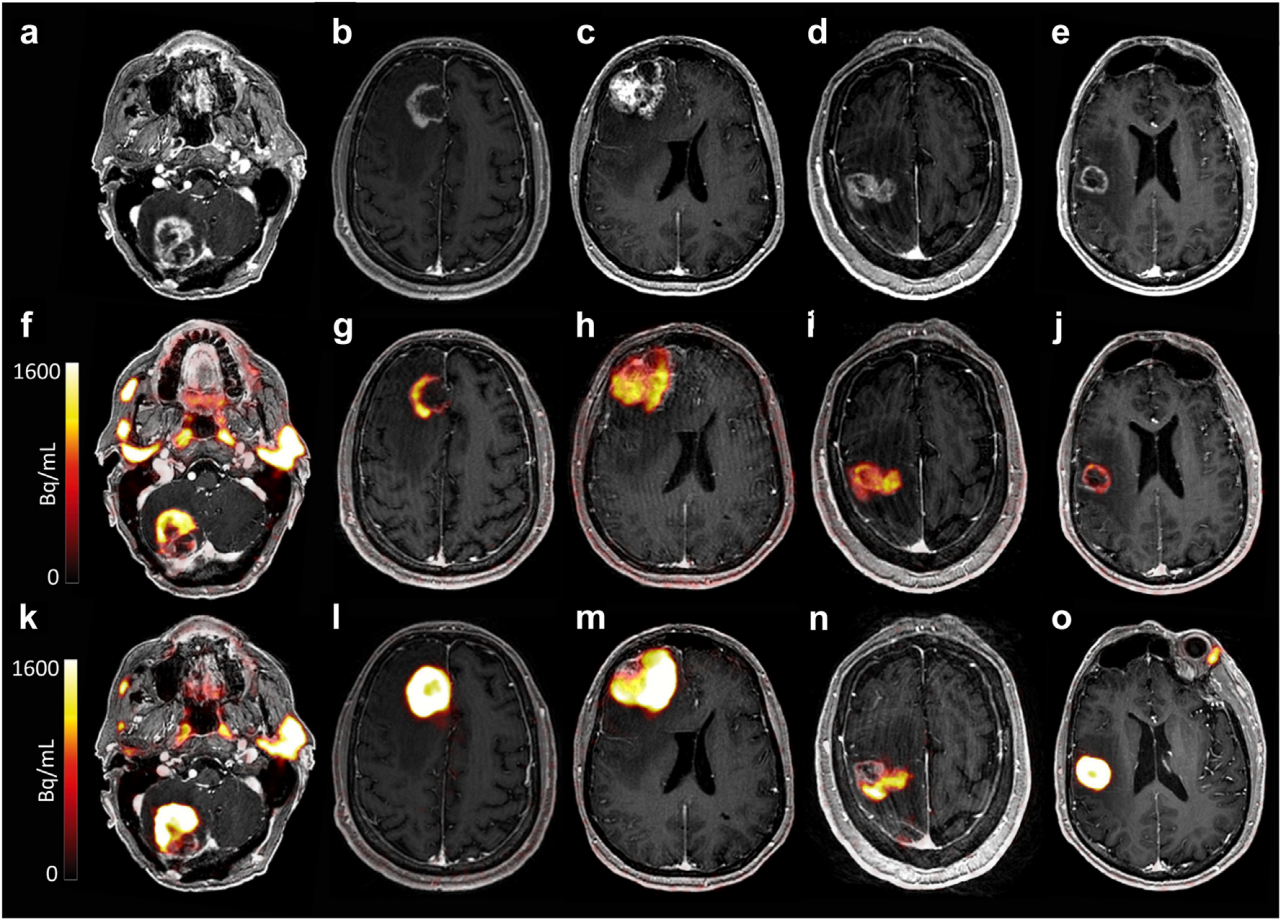
Uniformly scaled representative images of each patient with BM (from left to right, no. 6–10) including a post-contrast T1w MR image (a–e), and fused [^68^Ga]Ga-PSMA-11 PET with post-contrast T1w MR images after IV (f–j) and ssIA administration (k–o). Abbreviations: BM: brain metastasis; IV: intravenous; PSMA: prostate-specific membrane antigen; ssIA: super-selective intra-arterial.

All patients showed positive focal uptake of [^68^Ga]Ga-PSMA-11 at the tumour site both after IV and ssIA administration. Representative PET-MR images are shown for each patient with HGG (Fig. 3) and BM (Fig. 4). Visual inspection showed higher uptake after ssIA administration compared to IV administration in all patients.

**Fig. 5:**
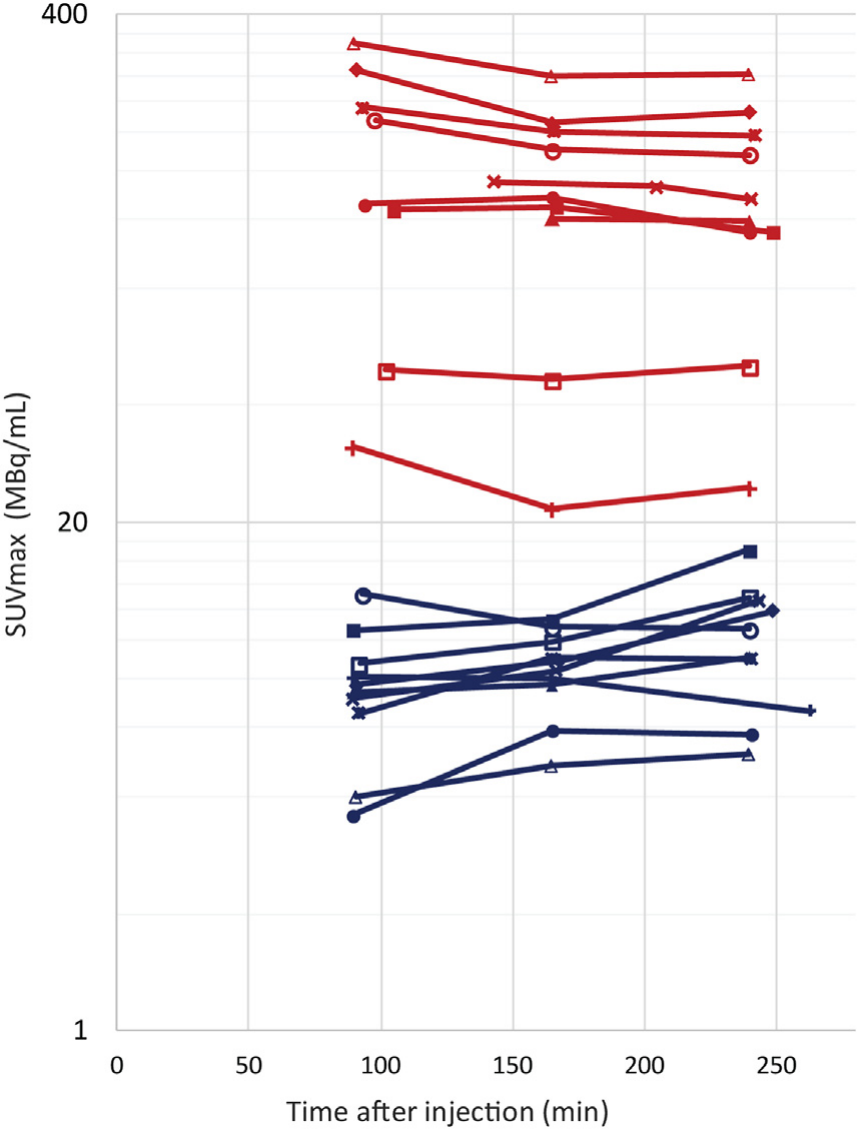
Time activity curves of SUVmax (MBq/mL) of [^68^Ga]Ga-PSMA-11 uptake in tumour for each patient (n = 10, each marker icon represents one patient) after IV (blue lines) and ssIA (red lines) administration of [^68^Ga]Ga-PSMA-11. Abbreviations: IV: intravenous; PSMA: prostate-specific membrane antigen; SUVmax: maximum standardised uptake value; MBq: megabecquerel; ssIA: super-selective intra-arterial.

Semi-quantitative analyses per patient based on the SUVmax across all time-points shown in Table 3 confirmed that ssIA administration resulted in a median 15-fold (IQR: 7–25 fold) higher uptake at the tumour site compared to IV administration (Fig. 5). According to the bootstrap analysis, the mean SUVmax after ssIA administration (168.8, 95% CI: 110.6–227.0) was well beyond the estimated 95% CI after IV administration (10.5, 95% CI: 8.4–12.7; Table 4).

**Table 3:** [^68^Ga]Ga-PSMA tumour uptake after intravenous and super-selective intra-arterial administration.

	IV administration							ssIA administration						
	Scan time p.i. (min)^a^	SUVmax	TBRmax^b^	SG SUVmean	T/SG ratio	Liver SUVmean	T/L ratio	Scan time p.i. (min)^a^	SUVmax	TBRmax^b^	SG SUVmean	T/SG ratio	Liver SUVmean	T/L ratio
1	90 165 241	3.6 5.9 5.8	359 – –	12.4 15.4 17.4	0.4	6.0	1.0	94 165 240	131.0 136.0 111.0	– – –	13.0 15.7 15.8	9.2	5.0	27.4
2	92 165 241	6.5 9.0 9.0	– – –	12.0 12.9 13.2	0.7	6.1	1.5	93 166 242	231.7 200.4 196.7	3 604^c^ 2 671 6 116	19.0 21.9 21.9	11.1	9.1	25.5
3	90 165 240	7.3 7.7 9.1	– – –	18.8 18.9 21.3	0.5	8.7	1.0	90 165 240	n.a. 120.2 118.2	n.a. – 10 626	22.2 24.6 23.3	5.2	7.5	16.1
4	90 165 263	8.1 8.0 6.6	269 265 329	18.1 19.4 20.7	0.4	3.9	2.0	91 165 240	31.3 21.7 24.6	988 1 027 2 328	17.8 17.6 18.1	1.8	4.0	7.9
5	91 167 245	7.1 8.4 12.7	710 840 1 269	11.0 11.6 12.8	1.1	5.2	2.5	143 205 241	149.5 145.0 135.0	13 071 12 680 11 803	10.6 11.9 12.6	12.8	6.5	23.0
6	90 165 240	10. 7 11.3 17.1	1 067 1 131 1 705	17.9 19.8 21.7	0.9	2.4	7.0	105 166 249	127.0 128.6 110.9	1 553 2 096 1 809	18.7 19.3 21.3	6.5	2.9	44.3
7	91 168 249	7.7 8.9 12.0	769 886 1 196	21.8 25.8 28.9	0.5	3.7	3.3	91 165 240	288.5 211.0 225.0	12 880 6 281 5 022	26.0 31.6 31.3	9.7	4.2	68.7
8	93 165 240	13.1 10.8 10.7	1 311 1 080 1 070	29.7 34.7 35.1	0.4	7.5	1.8	93 165 240	215.2 180.4 175.5	10 152 5 674 8 279	26.0 29.2 30.4	7.6	7.3	29.7
9	92 165 240	8.7 10.0 13.0	290 995 1 291	23.8 28.4 32.3	0.5	6.7	1.9	102 165 240	49.1 46.6 50.5	882 1 046 1 133	18.4 23.7 26.4	2.2	6.8	7.4
10	91 165 240	4.0 4.8 5.1	397 478 171	13.2 14.8 15.4	0.4	6.0	0.9	90 165 240	336.8 277.6 282.7	30 191 – –	17.4 18.6 17.5	18.9	6.4	52.8

Abbreviations: IV: intravenous; n.a.: not available; p.i.: post-injection; SG: salivary glands; ssIA: super-selective intra-arterial; TBR: tumour-to-background ratio; T/L: tumour-to-liver; T/SG: tumour-to-salivary glands (SUVmax tumour/pooled SUVmean parotid glands across time-points (t1-t3).

a After the first scan of the brain a static whole-body scan was also made (∼105 min p.i.).

b The TBRmax was calculated by the SUVmax of tumour divided by the SUVmean of background. For patients with no measurable uptake in background (SUVmean <0.01) no ratio was calculated (-).

c As a second lesion was observed in the contralateral cerebellum, to obtain a similar-sized VOI in healthy brain an ipsilateral cranio-caudal instead of left-right approach was taken and was placed in the cerebrum.

**Table 4:** Pooled data on [^68^Ga]Ga-PSMA-11 PET uptake and biodistribution after IV and ssIA administration in n = 10 patients.

	IV administration	ssIA administration	Mean Difference (95% bootstrap CI)^c^
**SUVmax**	**10.5 (7.5**–**13.0)**	**142.8 (102.8**–**245.9)**	158.3 (100.3–222.4)
t1	7.5 (5.9–9.2)	149.4 (88.2–260.1)	
t2	8.6 (7.2–10.2)	140.6 (101.8–203.1)	
t3	9.9 (6.4–12.7)	126.6 (95.8–203.7)	
**TBRmax**^a^	1 233 (425–1 607)	6 080 (1 583–8 673)	4 703 (1 443–8 126)
**SUVmean liver**	6.0 (3.9–6.9)	6.4 (4.1–7.3)	0.3 (−0.3 to 1.1)
**T/L-ratio**	1.8 (1.0–2.7)	26.5 (14.0–46.4)	28.0 (18.1–39.9)
**SUVmean SG**^b^	19.5 (14.0–26.2)	20.3 (17.1–24.6)	0.8 (−1.7 to 3.3)
**T/SG-ratio**	0.5 (0.4–0.7)	8.4 (4.4–11.5)	7.9 (5.1–11.1)

Data are represented as median (IQR).

Abbreviations: CI: confidence interval; IV: intravenous; IQR: interquartile range; RLT: radioligand therapy; SG: salivary glands; SUV: standardised uptake value; ssIA: super-selective intra-arterial; t: time-point; T/L: tumour-to-liver; T/SG: tumour-to-salivary glands.

a The TBRmax was calculated by the SUVmax of tumour divided by the SUVmean of background.

b For the SUVmean of both parotid glands (left and right) the data were pooled over the three time points post-injection (90, 165 and 240 min).

c Paired sample t-test mean differences with bootstrap 95% CI were reported.

In two patients (no. 4 and 9) ssIA administration showed less high uptake at the tumour site compared to the other patients. In patient no. 4 arteriovenous shunting was observed on DSA (Table 2). In patient no. 9, DSA showed preferential flow beyond the tumour, indicating potential venous vascularization.

Between tumour types (HGG versus BM), the difference in SUVmax after ssIA administration compared to IV administration was similar (respectively, 124.8, 95% CI: 61.1–179.7 and 191.9, 95% CI: 92.4–299.2).

In two patients with multiple BM, an intra-patient inter-lesion comparison of the selectivity of ssIA was possible. In one patient (no. 2) a new lesion in the contralateral cerebellum was found on the first series of PET-MRI scans, which was not yet present on the MRI performed for routine clinical care two weeks earlier. In this patient, the SUVmax was 26-fold higher after ssIA in the selectively approached lesion in the right cerebellar hemisphere (Table 3), compared to less than three-fold higher (5.1 versus 13.6) in the contralateral lesion that was not selectively approached. The other patient (no. 7) had one lesion in the frontal and one in the temporal lobe; of which only the larger frontal lesion was selectively approached. The frontal lesion showed a 24-fold higher SUVmax after ssIA, the temporal lesion showed no difference (4.4/5.1) (Table 3).

From the fused PET-MR images, in all patients [^68^Ga]Ga-PSMA-11 uptake showed to correspond with the area of contrast-enhancement on MRI and no uptake was seen outside these areas both after ssIA and IV administration (Supplementary Figure S2).

### Physiological uptake of [^68^Ga]Ga-PSMA-11

Almost negligible uptake was observed in tumour-surrounding and contralateral healthy brain (i.e., background), both after ssIA and IV administration (SUVmean range <0.1–0.1). After ssIA administration, the TBRmax (5 796, 95% CI: 2 588–9 663) was higher and well beyond the 95% CI for the TBRmax after IV administration (1 093, 95% CI: 710–1 416) (Table 3, Table 4).

Visual assessment of uptake in the remainder of body showed moderate to high uptake in salivary glands (90–240 min p.i.), liver, kidneys and bladder, and low to moderate uptake in spleen and bowel structures (all on whole-body PET, 90 min p.i.), with no marked difference between the scans following ssIA and IV administration for all patients. A large variability in parotid gland uptake was observed within and between patients (Table 4). Semi-quantitative analyses confirmed no difference in parotid gland uptake between ssIA and IV administration (SUVmean, 20.7, 95% CI: 17.5–24.1 and 20.0, 95% CI: 16.4–24.4; Table 4). Over time, the parotid glands showed almost stable retention between 90 and 240 min p.i., irrespective of the administration route (Supplementary Figure S3). Also, liver uptake showed no difference between ssIA and IV administration (SUVmean, 6.0, 95% CI: 4.8–7.0 and 5.6, 95% CI: 4.5–6.7). The mean T/SG and T/L ratios after ssIA administration (T/SG 8.5, 95% CI: 5.5–11.8; T/L 30.3, 95% CI: 18.8–42.2) were therefore well beyond the 95% CI after IV administration (T/SG 0.6, 95% CI: 0.4–0.7; T/L 2.3, 95% CI: 1.4–3.5; Table 4).

### Potential of PSMA-based RPT

Dosimetric modelling for [^177^Lu]Lu-PSMA-based RLT showed that the maximum absorbed radiation dose per tumour per treatment cycle was a median 5-fold higher (IQR: 2–24) after ssIA administration (68 Gy, IQR: 33–110) compared to IV administration (10 Gy, IQR: 6–26), and therefore well beyond the estimated 95% CI after IV administration (86.8, 95% CI: 46.8–133.9 versus 14.7, 95% CI: 8.3–21.9).

For [^225^Ac]Ac-PSMA-based RLT, the maximum absorbed radiation dose per tumour per treatment cycle was a median 3-fold higher (IQR: 2–15) after ssIA administration (78 Gy, IQR: 38–142) compared to IV administration (18 Gy, IQR: 11–40), and therefore well beyond the estimated 95% CI after IV administration (85.2, 95% CI: 50.6–117.7 versus 23.5, 95% CI: 13.9–33.8).

### PSMA expression in tumour biopsies

Post-operative tissue analysis of samples obtained from areas with [^68^Ga]Ga-PSMA-11 uptake on PET, confirmed high PSMA expression on endothelial cells of microvasculature and, to a lesser extent, on tumour cells of all patients (Supplementary Figure S4).

## Discussion

In this prospective clinical imaging study the feasibility, safety and benefit of ssIA (compared to IV) administration of a PSMA-targeting radioligand was assessed, with the aim to provide quantitative evidence and modelled predictions for the potential of PSMA-based RLT for treatment of malignant brain tumours. By means of hybrid PET-MRI, we showed uptake of [^68^Ga]Ga-PSMA-11 at the tumour site in all patients with HGG and BM (n = 5 and 5, respectively), and stable retention up to (at least) 240 min post-injection. A median 15-fold higher uptake was observed after ssIA administration, resulting in higher tumour to non-target uptake ratios, compared to IV administration. Dosimetric modelling showed that RLT, which usually includes at least four treatment cycles, has the potential to deliver high cumulative radiation doses locally to brain tumours, particularly through ssIA administration.

Uptake of the PSMA-based radioligand at the tumour site was expected to occur, given positive results from the use of PSMA as target in prostate cancer, and the observed high expression of PSMA on tumour microvasculature and to a lesser extent on tumour cells of HGG and BM.^4^ In vitro experiments demonstrated high affinity binding of PSMA-ligands with an equilibrium dissociation constant (K_D_) in the low nanomolar range.^34^ In vivo kinetic modelling studies in patients with prostate cancer showed that SUV of [^68^Ga]Ga-PSMA-11 strongly correlates with the net influx rate (constant Ki).^35^ We therefore hypothesized that ssIA administration, generating a very high peak radioligand concentration, would increase uptake at the tumour site.

While uptake at the tumour site was a median 15-fold higher after ssIA administration compared to IV administration, uptake in healthy brain was comparable and negligible, which is a prerequisite for safe RLT. Radiation exposure to the remainder of body (i.e., healthy organs), was also nearly equal after ssIA and IV administration. This is likely due to the fact that, as generally the case in RLT strategies, the cumulative uptake of radioligand in tumour is only a small percentage of the total injected activity. The remainder activity will distribute alike with IV administration, thus potential differences less likely occur or become evident. And, given the relatively small tumour volume (i.e., PSMA-positive load) in brain, the “sink effect” will not occur, as this has been shown only to occur in patients with very high tumour loads (i.e., diffusely metastasized prostate cancer, with total PSMA-positive tumour load >1355 mL).^36^ Considering that thresholds for treatment usually take into account target to non-target ratios, we showed that the ssIA approach significantly increases the efficiency of RLT by widening the potential therapeutic window, therewith qualifying more patients for therapy.

The salivary glands, which are considered the dose-limiting organs for PSMA-based RLT, showed generally high tracer uptake (maximum SUVmean of ∼33), with large variability within and between patients. This is in accordance with prostate cancer literature,^19^ and initiatives are ongoing to identify ways to decrease salivary gland uptake or to restore salivary function (NCT04593589). Importantly, with IV administration tumour uptake exceeded salivary gland uptake in only one out of ten patients (T/SG ratio 1.1, median 0.5), corresponding to what was observed in a first clinical series by Truckenmueller et al.^6^^,^^11^ With ssIA administration, T/SG uptake ratios were higher, up to 18.9 (median 8.4). This was not only statistically-significant, but also clinically relevant as this qualifies all (10/10) patients for PSMA-based RLT (versus only 6/10 after IV administration) based on EANM guidelines using a T/SG ratio threshold of 0.5.^37^ While liver uptake was only measured once (scan 1), we expect this to decrease beyond 90 min p.i. based on available literature, therewith assuming also favourable T/L ratios.^38^^,^^39^ As potential pitfall for patient selection, it should be noted that (treatment-related) inflammation may also cause uptake of PSMA-based radioligands, as shown in patients with varying diseases (including nine glioblastoma cases) and rats using [^68^Ga]Ga-PSMA and [^18^F]F-DCFPyl.^40^, ^41^, ^42^ Future studies should validate these findings in patients with brain tumours and which, for instance MRI, parameters may guide differentiation and patient selection.

Furthermore, research is needed to acquire knowledge on the radiobiological effects of RLT in patients with malignant brain tumours. From extensive clinical experience with external-beam radiation therapy (EBRT) it is known that brain tumours are sensitive to radiation.^43^ The dose delivery rate to the tumour, however, is different between EBRT and RLT.^44^ Given the high expression of PSMA on endothelial cells of tumour microvasculature and to a lesser extent on tumour cells, future ssIA-PMSA-based RLT will likely be most effective using beta-emitting radioligands, such as lutetium-177, that are characterised by a longer path length (0.05–12 mm) compared to alpha-emitters (40–100 μm). When alpha- or auger-emitting PSMA-based radioligands are used, which benefit from a higher linear energy transfer, the radioligand-complex will need to be transported from the endothelial cells into the tumour microenvironment to reach the tumour DNA, such as occurs in prostate cancer.^45^ It is unknown yet whether this also occurs in brain tumours.^46^ Moreover, it may be the case that PSMA expressing tumour cells will not be reached by radiation in areas where the BBB is intact. For future studies we therefore also consider ongoing technical advancements, such as strategies to circumvent the BBB (e.g., convection enhanced delivery), or to temporarily enhance BBB permeability (e.g., MRI guided focused ultrasound).^47^^,^^48^

To date, three case reports^8^, ^9^, ^10^ and one small case study (n = 3)^6^^,^^11^ have reported on the use of [^177^Lu]Lu-PSMA-based RLT for patients with HGG, with administered activities up to 8390 MBq via conventional IV administration. Although long-term outcome of these patients is unknown, no treatment related toxicities,^6^^,^^10^ improved quality of life,^9^ and a significant decrease in post-treatment [^68^Ga]Ga-PSMA uptake (suggesting treatment effect) were reported.^10^ The reported maximum absorbed dose to tumour varied significantly from 0.4^11^ up to 14 Gy.^8^ Two case reports^13^^,^^14^ and one case study (n = 3)^12^ have reported on the use of either [^177^Lu]Lu-PSMA or [^225^Ac]Ac-PSMA-based RLT in patients with BM, with administered activities up to 6000 MBq and 8 MBq, respectively, via IV administration. Also here the long-term outcome of patients is unknown, yet a significant decrease in [^68^Ga]Ga-PSMA uptake was reported.^13^^,^^14^ Xerostomia was the most common side-effect and denoted as intolerable for activities exceeding 100 kBq/kg of [^225^Ac]Ac-PSMA-617.^12^ In our cohort, one patient experienced semi-acute partial weakness during the first 24 h after ssIA administration, but no causal relation was found in an extensive stroke work-up and the patient fully recovered within a few days without any medical intervention. The observed acceptable safety profile is in accordance with widely available literature on diagnostic angiographies, reporting generally low morbidity of intracerebral (non-selective) IA procedures.^17^

This study had several limitations and potential confounding factors to consider. Firstly, it concerned a single-centre study with limited patient numbers, as a result of the relative complexity, high burden and lack of benefit for participating patients. No randomisation was applied, because each patient underwent both procedures. Theoretically, these factors had the potential for confounding the results. Furthermore, dosimetric modelling (performed according to the EANM/MIRD guidelines^23^) included a number of assumptions regarding binding profiles and biokinetic behaviour of [^177^Lu]Lu-PSMA and [^225^Ac]Ac-PSMA, based on the observed uptake kinetics of [^68^Ga]Ga-PSMA-11 and extrapolation beyond 240 min p.i. Future dosimetry studies using radionuclides with a long (er) half-life, such as zirconium-89,^49^ can substantiate these assumptions.

To conclude, we show, supported by quantification, the potential of PSMA as target for RLT in patients with brain tumours, and the benefit of ssIA compared to IV administration. Our results justify further studies into the concept of ssIA administration, which could include radioligands but also chemotherapeutics or immunomodulators. A next study on RLT for patients with malignant brain tumours will first be directed at establishing the maximum tolerated dose and minimum treatment interval time (phase I), followed by preliminary efficacy (phase II) of ssIA-PSMA-based RLT, likely using lutetium-177, initially adjuvant to existing strategies, and subsequently combined with other strategies to optimise radioligand uptake at the tumour site.

## Contributors

IJP, PJD, MJB, FAV, MSm and SEMVZ contributed to the study conception and design. Material preparation, data collection and analysis were performed by IJP, PJD, RKB, AAH, LCJ, MK, MSe, RV, and SEMVZ. The first draft of the manuscript was written by IJP and SEMVZ and all authors (IJP, PJD, RKB, MJB, AAH, LCJ, MK, MSe, RV, FAV, MSm and SEMVZ) reviewed and edited previous versions of the manuscript. The underlying data was accessed and verified by IJP and SEMVZ. All authors read and approved the final manuscript.

## Data sharing statement

The individual participant data that underlie the results reported in this article, after de-identification (text, tables, figures, and appendices), will be available immediately following publication to investigators whose proposed use of the data has been approved by an independent review committee identified for this purpose and to achieve aims in the approved proposal, upon reasonable request and after signing a data access agreement.

## Declaration of interests

PJD received speaker honoraria (paid to institution) from Stryker and Siemens, and is consultant to Philips (paid to institution). FAV received speaker honoraria (paid to employer) from Sanofi, AstraZeneca, Bayer, and is consultant (paid to employer) to GE Healthcare. MSm received speaker honoraria (paid to employer) from Auntminnie and GE Healthcare and consultancy fees (paid to institution) from Bracco.
