# Supplementary material for: Potential of PSMA-targeting radioligand therapy for malignant primary and secondary brain tumours using super-selective intra-arterial administration: a single centre, open label, non-randomised prospective imaging study
Source: eBioMedicine. 2024 Mar 22;102:105068. doi: 10.1016/j.ebiom.2024.105068 (PMC10981001; doi:10.1016/j.ebiom.2024.105068)
Supplement: Supplementary Figures [file mmc1.pdf]

Supplementary Data

Contents

Contents 1
Safety Monitoring Charter 2
Supplementary Figures 5

Safety Monitoring Charter

Introduction

The purpose of this charter is to define the responsibilities of the independent medical monitor, give detailed membership requirements, describe the data to be reviewed, delineate the reception and reviewing process, and outline considerations and policies of the medical monitor. The medical monitor will act in an expert independent advisory capacity to monitor patient safety.

Study overview

- Title: PSMA-PET in intra-axial brain tumors
- Sponsor: Erasmus Medical Center, Rotterdam
- Design: non-randomized, single center, prospective imaging proof-of-concept study
- Number of participants: 15-25 patients will be included for intravenous injection of [^{68}Ga]Ga-PSMA-11 and a PET/MRI scan. From this group, we expect in at least 10 patients a positive uptake signal at the tumor site, making them eligible for intra-arterial injection of [^{68}Ga]Ga-PSMA-11 and a second PET/MRI scan (Figure 1).

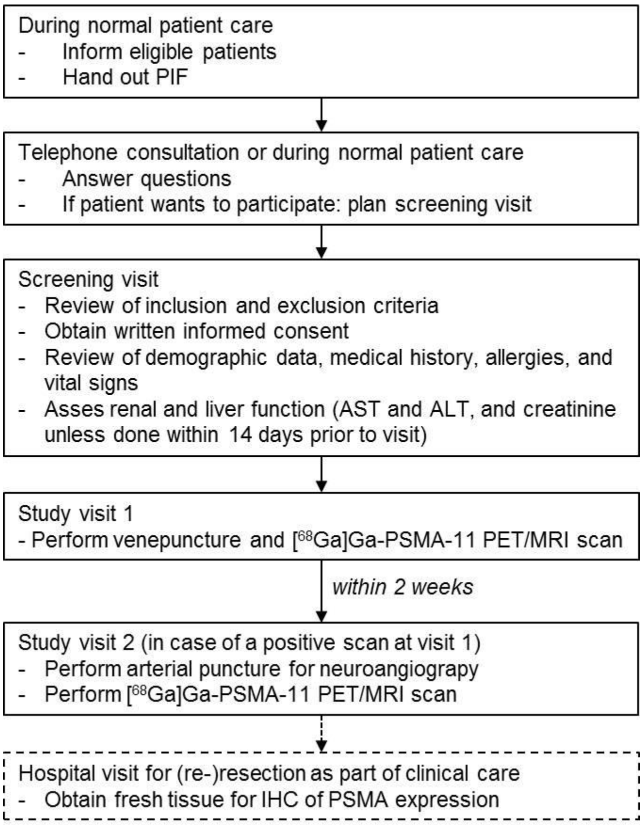

Figure 1. Flow chart of the study.

Roles and responsibilities

The aim of the medical monitor is to defend the interest of patients enrolled in this study and to evaluate the safety of the investigational procedures used in this study. Specifically, the medical monitor will be responsible for conducting interim evaluations concerning the following:

- Adverse events (AEs), serious adverse events (SAEs), serious adverse reactions (SARs) and suspected unexpected serious adverse reactions (SUSARs)
    - Evaluate events that occur during, and between the study visits
    - Individual events of particular concern
    - Any other safety-supporting data requested by the medical monitor
- Make a short report of the safety of the procedures of the study after every patient in case of any concern for the Principal Investigator (PI). The PI will then decide if continuation of the inclusion of patients is safe. This decision does not lie with the medical monitor.

Interim evaluation

The medical monitor will get unblinded data on all AEs, SAEs, SARs and SUSARs. The medical monitor will evaluate if there is a significant occurrence of AEs, SAEs, SARs and SUSARs during and between the two study visits for every patient included.

Protocol availability

The potential medical monitor will have access to the protocol before agreeing to monitor the study.

Composition

The medical monitor for this study is:

Prof. dr. C.B.L.M. Majoie

Department of Radiology & Nuclear MedicineAmsterdam UMC, location AMC

Relationships

The medical monitor is independent of the study and was not involved in the creation of the protocol and has no competing interests. The medical monitor will have to sign a competing interests form.

Organization of medical monitor data reporting

If after one of the included patients the medical monitor concludes the safety of the patient is compromised or, after evaluation the medical monitor believes that safe continuation of the study cannot be guaranteed he will report this to the Radiology and Nuclear Medicine Trial Bureau at Erasmus MC. All blinded data needed for the evaluation per patient will be provided by the investigator. The conversion code to unblind the data will be provided by personnel from the Trial Bureau. There is no need for a (annual) meeting.

Trial documentation and procedures to ensure confidentiality and proper communication

The medical monitor will not be blinded and will have access to all study data. The medical monitor does not have the right to share confidential information with anyone else, including members of the study team. The medical monitor will report in writing to the PI. Confidential papers and data should be destroyed by the medical monitor after reporting to the PI.

Supplementary Figures

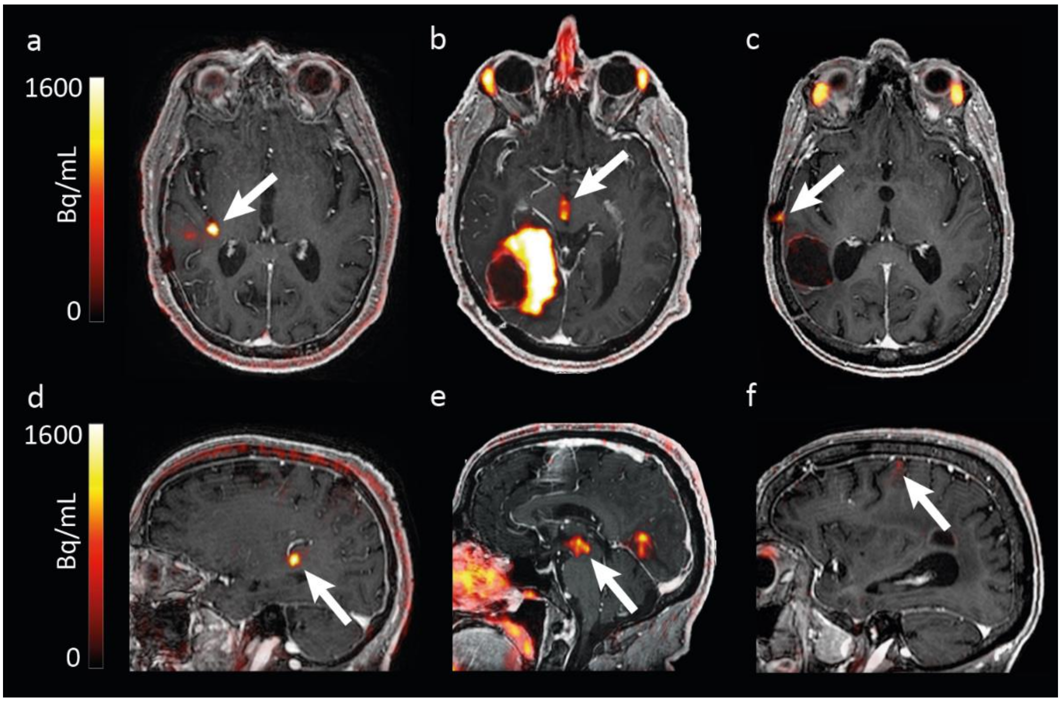

Supplementary Figure 1. Fused axial (a-c) and sagittal (d-f) PET with post-contrast T1w MR images of patient no. 1, 3 and 4 (from left to right) after ssIA administration (i.e., 240 min p.i.) highlighting the observed [^{68}Ga]Ga-PSMA-11 in brain/-vasculature at location of the catheter tip (white arrow). Abbreviations: MR: magnetic resonance, PET: positron emission tomography, p.i.: post-injection, PSMA: prostate-specific membrane antigen, ssIA: super-selective intra-arterial, T1w: T1-weighted.

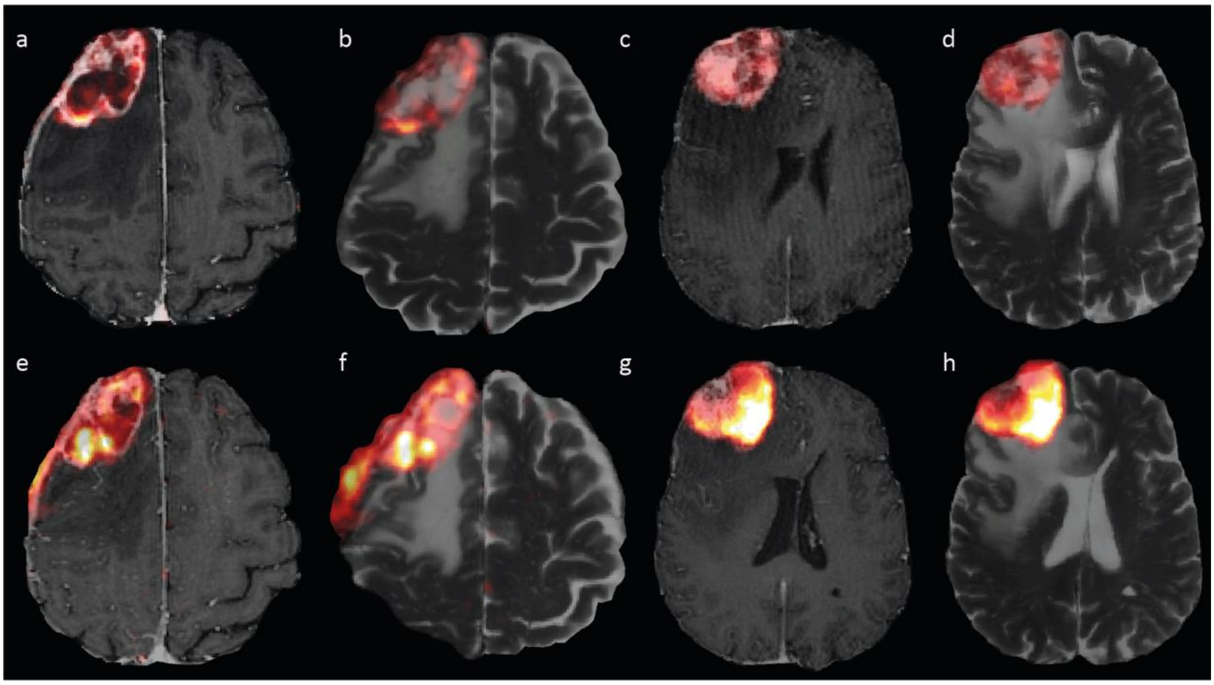

Supplementary Figure 2. Uniformly scaled representative images of two patients with HGG (no. 5, a, b, e, f) and BM (no. 8, c, d, g, h) including fused PET with post-contrast T1w MR (a, e, c, g) and T2w MR (b, f, d, h) after IV (a-d) and ssIA (e-h) administration. [^{68}Ga]Ga-PSMA-11 uptake corresponds with the area showing contrast-enhancement on MRI and no uptake was seen outside these areas both after ssIA and IV administration. Abbreviations: BM: brain metastasis, HGG: high-grade glioma, MR: magnetic resonance, PET: positron emission tomography, PSMA: prostate-specific membrane antigen, ssIA: super-selective intra-arterial, T1w: T1-weighted, T2w: T2-weighted.

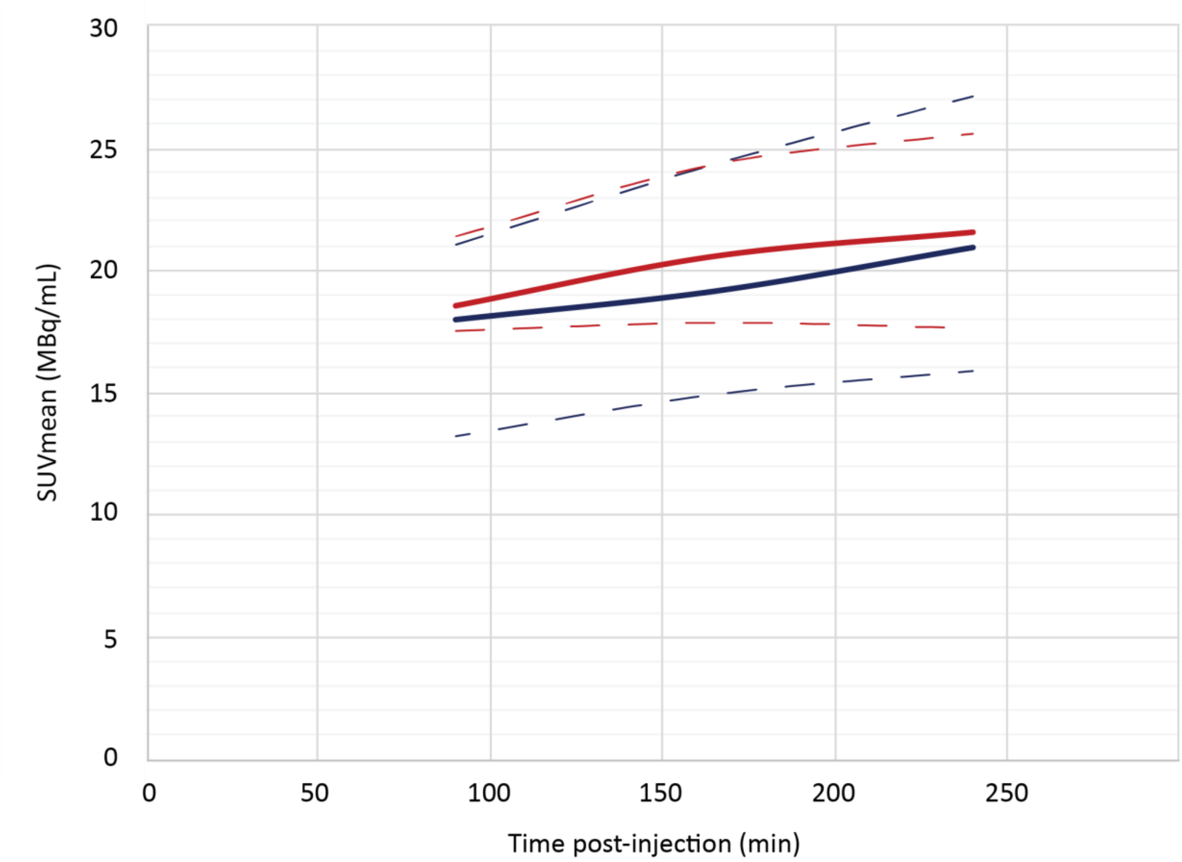

Supplementary Figure 3. Time activity curves of [^{68}Ga]Ga-PSMA-11 uptake in parotid glands (values left and right parotid were pooled) visualised as median (solid line, IQR in dashed lines) SUVmean (MBq/mL) pooled for all patients (n=10) after intravenous (blue) and super-selective intra-arterial (red) administration. Abbreviations: IQR: interquartile range; PSMA: prostate-specific membrane antigen; SUV: standardised uptake value; MBq: megabecquerel.

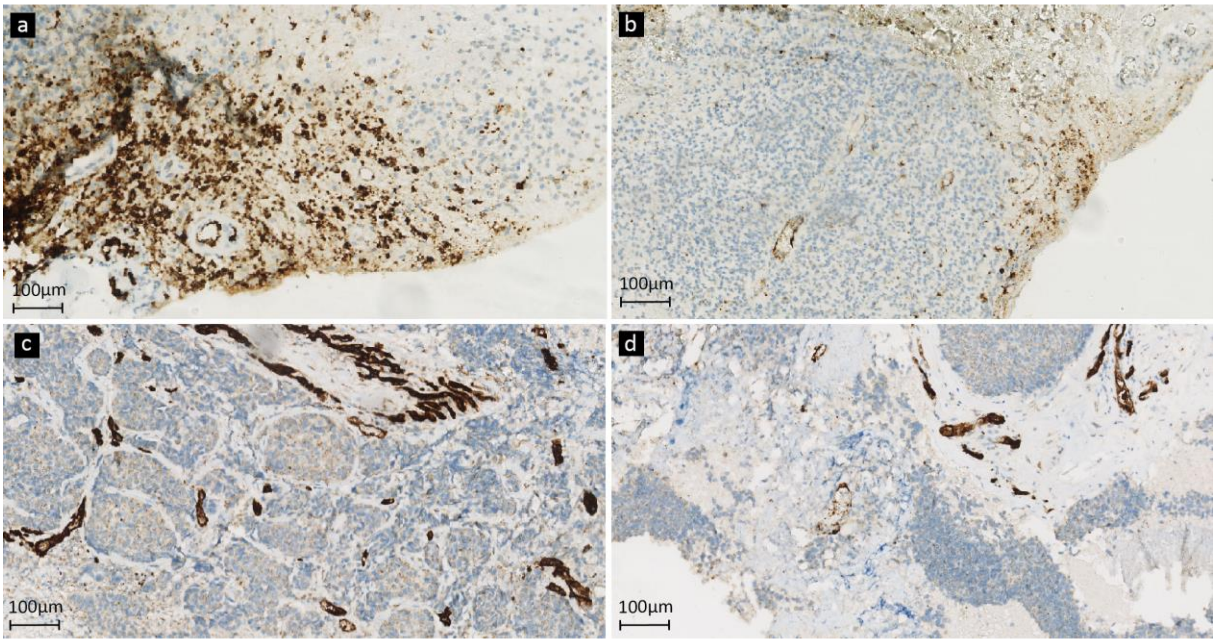

Supplementary Figure 4. Postoperative tissue analysis of representative samples of a patient with HGG (no. 5, a, b) and BM (no. 8, c, d) from areas with [^{68}Ga]Ga-PSMA-11 uptake on PET, showing extensive and strong PSMA expression (brown staining) on endothelial cells of microvasculature and, to a lesser extent, in tumour cells. Tumour cell dense areas are recognised by disorganised and dense arrangements of cell nuclei (blue staining). Abbreviations: BM: brain metastases, HGG: high-grade glioma, PSMA: prostate-specific membrane antigen.
